# Successful preoperative diagnosis and laparoscopic management of primary small bowel volvulus: A case report and literature review

**DOI:** 10.1097/MD.0000000000039391

**Published:** 2024-08-16

**Authors:** Seito Shimizu, Hitoshi Hara, Yasuhide Muto, Tomoki Kido, Ryohei Miyata

**Affiliations:** a Department of Surgery, Social Welfare Organization Saiseikai Imperial Gift Foundation Inc., Saiseikai Kazo Hospital, Kazo, Japan

**Keywords:** case report, laparoscopic surgery, preoperative diagnosis, primary small bowel volvulus.

## Abstract

**Rationale:**

Small bowel volvulus (SBV) is a rare cause of acute abdominal pain in adults, which requires surgical intervention to prevent small bowel necrosis. Primary SBV is rare, and its preoperative diagnosis is challenging. This report describes a case of primary SBV diagnosed preoperatively and treated laparoscopically.

**Patient concerns:**

A 56-year-old man presented complaining of sudden-onset abdominal pain of 3-hour duration. Physical examination revealed tenderness in periumbilical and upper abdominal regions with no signs of peritonitis.

**Diagnosis:**

Contrast-enhanced computed tomography revealed a 360°-clockwise rotation of the small intestine when viewed caudally at the mesenteric base. At this region, the superior mesenteric vein was interrupted. Although no dilation of the small intestine was observed, increased density in the mesentery of the affected area was observed. Minimal ascites was observed in the pelvic cavity. No evidence of congenital or secondary SBV was observed, supporting the diagnosis of primary SBV.

**Interventions:**

Laparoscopic detorsion of the SBV was performed approximately 6 hours after the onset of symptoms. No signs of bowel necrosis were observed, and the procedure was concluded after releasing the torsion.

**Outcomes:**

Severe abdominal pain disappeared immediately after surgery. The postoperative course was uneventful, and the patient was discharged on the 8th postoperative day.

**Lessons:**

This case highlights the importance of preoperatively diagnosing SBV, which enables early laparoscopic devolvulation without bowel resection.

## 1. Introduction

Small bowel volvulus (SBV) is a rare cause of acute abdominal pain in adults and necessitates surgical intervention to prevent small bowel necrosis. Primary SBV is SBV occurring without underlying medical conditions or anatomical abnormalities.^[1]^ Since SBV is a rare disease, preoperative diagnosis is challenging, and emergency exploratory laparotomy is often required for diagnosis.^[2]^ Even if it is diagnosed during diagnostic laparoscopy, devolvulation within the limited visual field is difficult, and conversion to laparotomy is frequently required.^[3]^ In this report, we present a case of primary SBV diagnosed preoperatively and treated laparoscopically.

## 2. Case presentation

A 56-year-old male presented to the emergency department complaining of sudden-onset abdominal pain of 3-hour duration. He had no relevant medical history and was not taking any regular medication. During his youth, he was a judo athlete with a body weight exceeding 90 kg. At the time of admission, his height, weight, and body mass index were 174 cm, 67 kg, and 22.1 kg/m^2^, respectively. His body weight had decreased by over 20 kg since adolescence. On examination, the vital signs were within the normal ranges, and the patient was afebrile. He had intense abdominal pain and tenderness in the periumbilical and upper abdominal regions with no signs of peritonitis.

Biochemical analyses revealed an elevated level of lactate dehydrogenase (293 U/L). Renal and hepatic function tests were normal, and no increase in the levels of inflammatory markers was observed.

**Figure 1.**
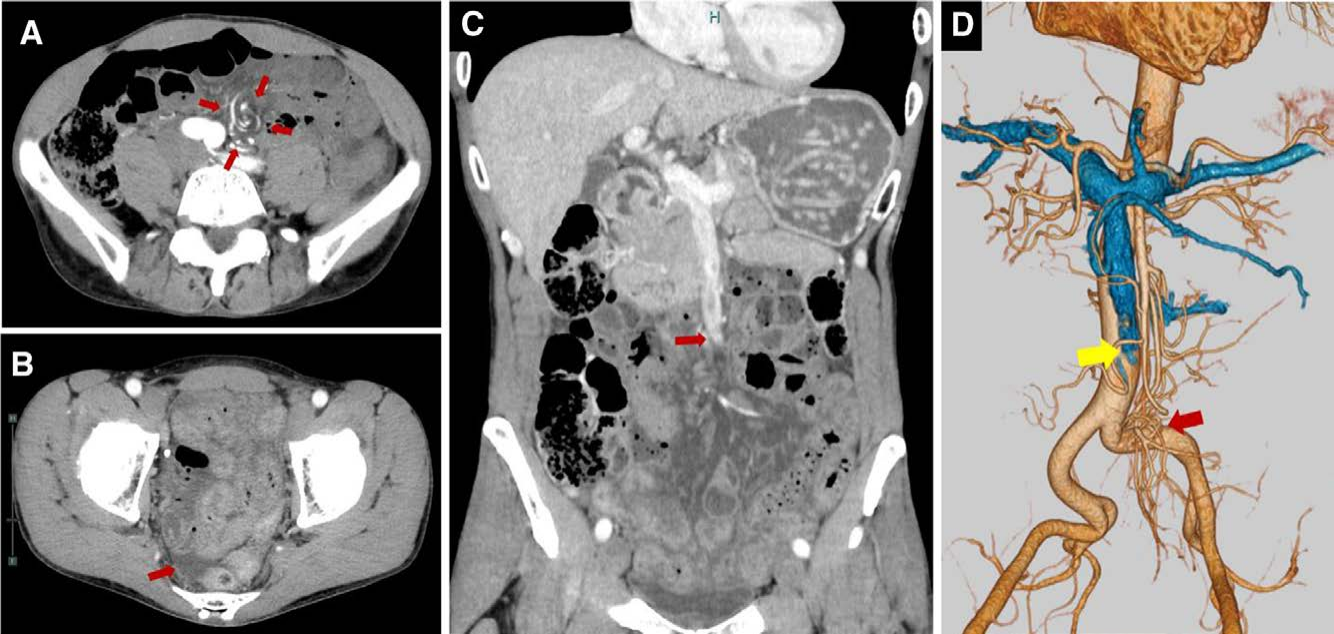
Preoperative contrast-enhanced computed tomography findings. (A) When the mesenteric base is viewed caudally, a 360°-clockwise rotation of the mesentery of the small intestine characterized by the rotation of branches of the superior mesenteric artery (SMA), known as the “whirl sign,” is revealed (red arrows). (B) Minimal ascites is present in the pelvic cavity (red arrow). (C) The rotation of the mesentery of the small intestine has resulted in the occlusion of the superior mesenteric vein (SMV; red arrow). Edematous changes in the mesentery of the small intestine, characterized by increased fat density, are visible. No obstruction or dilation of the small intestine is observed. (D) Three-dimensional angiography reveals small intestinal mesenteric torsion resulting in the occlusion of the SMV (yellow arrow) and rotation of the SMA with maintained blood flow (red arrow).

Contrast-enhanced computed tomography (CT) revealed a 360°-clockwise rotation of the small intestine, known as the “whirl sign,” when viewed caudally at the mesenteric base (Fig. 1A). Minimal ascitic fluid was observed in the pelvic cavity (Fig. 1B). Although no dilation of the small intestine was observed, edematous changes in the mesentery of the small intestine, characterized by increased density in the mesentery of the affected area, were present (Fig. 1C). At this mesenteric torsion site, the rotation of the mesentery resulted in occlusion of the superior mesenteric vein (SMV), while the superior mesenteric artery remained patent (Fig. 1D). Findings were consistent with SBV. No evidence of congenital intestinal rotational abnormalities, mesenteric tumors, or adhesions (no history of abdominal surgery) that could cause axial torsion was present, supporting the diagnosis of primary SBV.

During the examination, the patient experienced a rapid alleviation of abdominal pain. Considering the potential spontaneous resolution of primary SBV, the patient was urgently admitted for observation. However, severe abdominal pain recurred on the evening of the admission day. Although the initial presentation suggested spontaneous resolution of SBV with symptomatic improvement, the incomplete resolution led to recurrence. Therefore, emergency surgery was deemed necessary. Laparoscopic devolvulation of the SBV was performed approximately 6 hours after the onset of symptoms.

**Figure 2.**
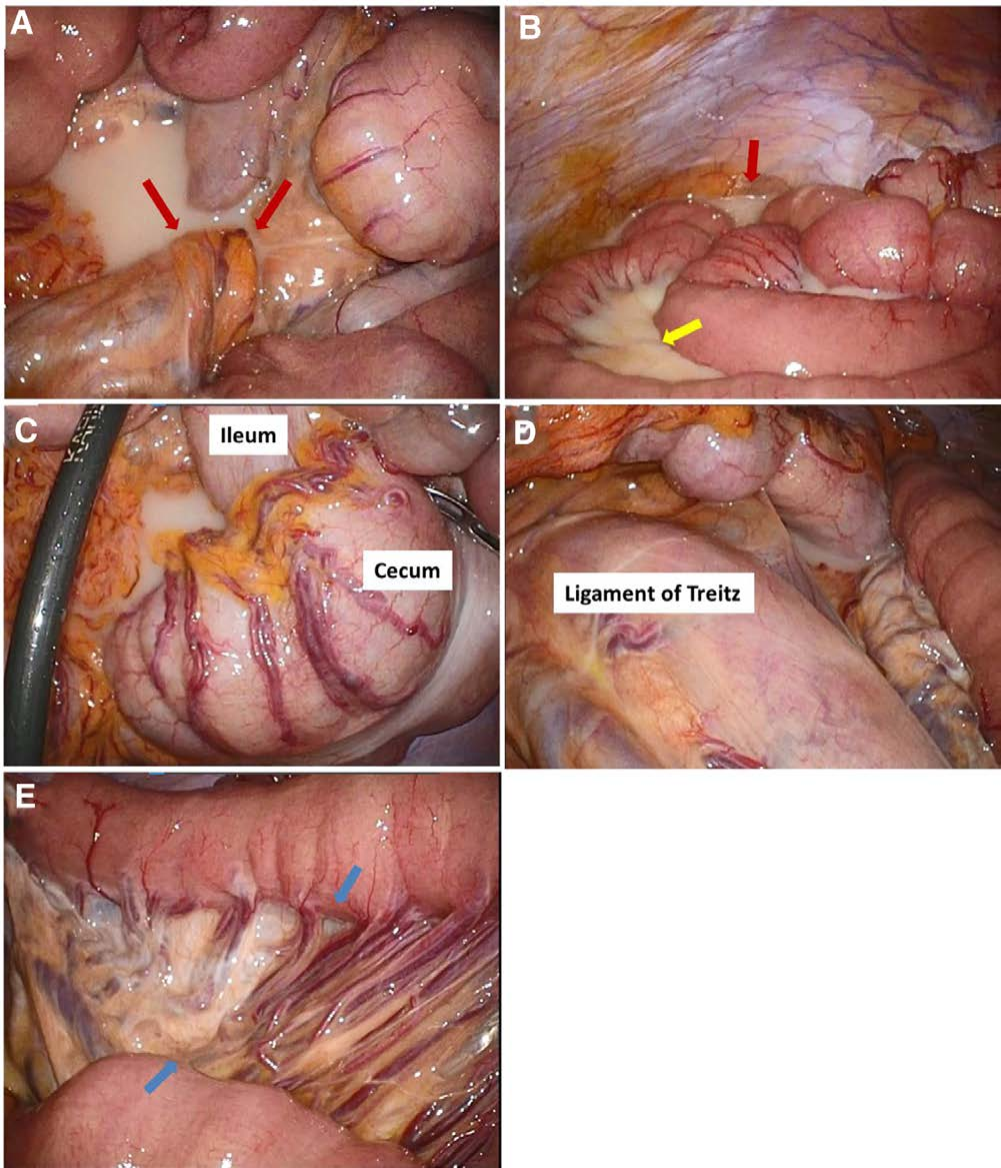
Laparoscopic findings. (A) Torsion is identified at the mesenteric base of the small intestine (red arrows). (B) Chylous ascites is observed in the abdominal cavity (red arrow). The mesentery of the small intestine appears whitish owing to lymphatic edema (yellow arrow). (C and D) The small intestine is sequentially pulled caudally from the terminal ileum to the ligament of Treitz, and the small bowel volvulus is released. (E) The boundary of lymphatic edema is observed in the mesentery of the upper jejunum, which suggests the starting of the rotation of small intestinal mesentery from the boundary (blue arrows).

Laparoscopic procedures were performed using a balloon-equipped camera port at the umbilicus and 2 additional 5-mm ports in the right upper and lower abdomen. Torsion of the mesenteric base of the small intestine was identified (Fig. 2A). Intraoperatively, moderate chylous ascites was observed (Fig. 2B). The mesentery of the small intestine appeared whitish owing to lymphatic edema (Fig. 2B). After identifying the ileocecal region, the small intestine was sequentially pulled caudally from the terminal ileum to the ligament of Treitz, which led to the sequential pulling of the mesentery from the anal to the oral side. Thus, the mesenteric torsion was released (Fig. 2C and 2D). Following devolvulation, a boundary of lymphatic edema was observed in the mesentery of the upper jejunum, suggesting that the anal side was twisted (Fig. 2E). The mesenteric fat was minimal to the extent that the mesenteric vessels were visible through it. No signs of bowel necrosis were observed, and the procedure was concluded after releasing the torsion. Anatomical abnormalities or coexisting diseases causing SBV were not evident, consistent with the diagnosis of primary SBV. The surgery was concluded without performing enteropexy.

The severe abdominal pain disappeared immediately after surgery. The postoperative course was uneventful. Diet was initiated on the 2nd postoperative day, and the patient was discharged on the 8th postoperative day.

## 3. Discussion

Adult-onset SBV is a rare cause of acute abdominal pain requiring surgery to prevent bowel necrosis. This report discusses a preoperatively diagnosed case of primary SBV treated laparoscopically.

In terms of etiology, SBV may be congenital, primary, or secondary. Volvulus is considered primary when it occurs without predisposing factors. Congenital SBV is associated with anomalies such as intestinal rotation abnormalities and incomplete mesenteric fixation, whereas secondary SBV arises from postoperative adhesions and anatomical abnormalities leading to twisting, such as abdominal tumors.^[1,4]^ Preoperative diagnosis of primary SBV is challenging, and emergency laparotomy is often required for diagnosing the condition. While reported cases of spontaneously resolving SBV exist,^[5]^ SBV essentially requires urgent surgical intervention. Even if diagnosed laparoscopically, unraveling the volvulus in a limited visual field is difficult, often necessitating conversion to laparotomy.

SBV often lacks specific findings, making preoperative diagnosis challenging.^[6]^ CT findings suggestive of SBV include the “whirl sign” and “barber’s pole sign,” which are indicative of torsion.^[7]^ Three-dimensional angiography is valuable for diagnosis since it reveals interrupted blood flow in the SMV due to torsion.^[2]^ While the intestines are distant from the center of rotation, the mesentery, being at the center, experiences significant torsion. Despite presenting with severe abdominal pain, SBV does not typically result in intestinal obstruction or signs of peritoneal irritation during its early stages. In addition, regarding the vascular structures within the twisted mesentery, veins and lymphatics are more fragile with low internal pressures compared with arteries. Consequently, venous and lymphatic flow disorders are likely to occur initially. The mesenteric lymphatic edema and chylous ascites observed in the twisted region reflect these pathophysiological changes, serving as characteristic features of the early stages of the disease.^[8]^ Prompt recognition of SBV and intervention are essential. Cases of spontaneously resolving SBV have been reported^[5]^; however, considering the possibility of bowel necrosis, surgical intervention is necessary when SBV does not completely resolve naturally. Although temporary relief from symptoms and natural resolution were initially anticipated, the symptoms recurred, necessitating surgical intervention.

In the absence of preoperative diagnosis, patients with SBV are often subjected to exploratory laparotomy. If diagnosed preoperatively, a strategic surgical approach based on the diagnosis is feasible. Prompt surgery is desirable before bowel necrosis occurs. In the early stages of SBV, an increased likelihood of preserving the intestine exists, and since bowel obstruction has not yet occurred, the establishment of a working space is possible, enabling minimally invasive laparoscopic surgery.^[8]^ Given that SBV involves torsion at the mesenteric base with a significant extent of twisted intestine for reduction, achieving a clear view with laparoscopy might be somewhat challenging. The small intestinal mesentery is arranged to fold from the distal to the proximal direction and from the dorsal to the ventral side.^[9]^ Thus, even in cases where visualizing the twisted region is difficult, as performed in the present case, tracing from the terminal ileum to the ligament of Treitz and pulling the mesentery caudally allow for anatomical devolvulation. Complete laparoscopic surgery may be challenging in cases in which bowel necrosis necessitates resection. In such cases, extending the umbilical port site through a small incision a few centimeters above the mesenteric base and lifting the entire small intestine out of the body can facilitate a direct view, ensuring effective detorsion and resection anastomosis of the intestine.^[3]^

We reviewed the previously published cases of primary SBV in adults using the PubMed database. Seven cases were identified, and thus, in total, we reviewed 8 cases, including the present case^[8,10–15]^ (median patient age, 65 years; males, 6; females, 2; and male-to-female ratio, 1:3; Table 1). The direction of rotation was clockwise in 1 case (the present case) and counterclockwise in 2 cases, with rotation angles of 360°, 180°, and 720°, respectively. Preoperative diagnosis of SBV was successful in 4 cases^[10–12]^ (50%), including the present case, indicating that preoperative diagnosis of SBV is challenging. Imaging findings, including the “whirl sign,” distended small intestine, and edematous changes in the mesentery, were present in all these 4 cases. However, discontinuation of the SMV was observed only in the present case. The duration from the onset of symptoms to surgery ranged from 6 hours to 2 days. The surgical approach involved laparoscopy in 2 cases (25%) and laparotomy in 6 cases (75%). Laparoscopic surgery was performed in the early stage in 2 cases (within 6 hours and 1 day of onset, respectively). Bowel resection was performed in only 1 case after 2 days from the onset of symptoms. Devolvulation of SBV alone was performed in 7 cases (87.5%), whereas enteropexy was performed in 1 case. To the best of our knowledge, the present report is the first to describe a case of primary SBV in which laparoscopic surgery was strategically performed based on preoperative diagnosis. In addition, the present case had the shortest duration from the onset of symptoms to surgery among the reported cases.

**Table 1:** Summary of reported cases of primary small bowel volvulus.

Year	Reference	Age, yr	Sex	Rotation angle	Direction of rotation	Preoperative diagnosis	Image findings	Time from onset to surgery	Surgical approach	Intestinal resection
2016	Islam et al^[10]^	55	M	NR	NR	SBV, small bowel obstruction	Whirl sign and distended small bowel	2 d	Open ileum resection and detorsion of SBV	Yes
2017	Hayama et al^[11]^	70	M	Counterclockwise	180°	SBV, intestinal ischemia	Whirl sign and edematous changes of mesentery	1 d	Open detorsion of SBV	No
2019	Tsang et al^[12]^	78	W	NR	NR	SBV	Whirl sign and distended small bowel	1 d	Open detorsion of SBV	No
2020	Agrawal et al^[13]^	60	M	Counterclockwise	720°	Small bowel obstruction	Distended small bowel	2 d	Open detorsion of SBV	No
2021	Leaning^[14]^	79	W	NR	NR	Small bowel obstruction	Intraperitoneal fluid and distended small bowel	1 d	Open detorsion of SBV	No
2022	Bouassida et al^[15]^	73	M	NR	320°	Small bowel obstruction	Whirl sign	1 d	Open detorsion of SBV and enteropexy	No
2023	Gupta et al^[8]^	32	M	NR	NR	Internal hernia	Internal hernia involving the small intestine	1 d	Laparoscopic detorsion of SBV	No
2024	Our case	56	M	clockwise	360°	SBV	Whirl sign, discontinuation of SMV, and edematous changes of mesentery	6 h	Laparoscopic detorsion of SBV	No

M = man, NR = not reported, SBV = small bowel volvulus, W = woman.

This case report has several limitations. If extensive bowel necrosis leading to abdominal contamination or findings indicative of secondary SBV, such as adhesions or mesenteric tumors, are identified intraoperatively, and the limited incision approach is inadequate, conversion to laparotomy is necessary. Devolvulation alone has been reported to carry a 30% recurrence risk.^[16]^ While some authors consider enteropexy effective for preventing recurrence, the risk of fistula formation associated with enteropexy has been reported.^[17]^ No consensus on the advisability of additional mesenteric fixation after untwisting is available, and the results of the literature review described in this report do not allow for a definitive conclusion.

## 4. Conclusion

Herein, we reported a case of preoperative CT diagnosis and laparoscopic management of primary SBV. Diagnosing and conducting surgery in the early stages of the disease not only enable the establishment of a working space for minimally invasive laparoscopic procedures but also offer the advantage of potentially avoiding bowel resection. This report underscores the procedures for laparoscopic devolvulation and the importance of preoperative diagnosis of SBV, particularly for enabling early interventions.

## Acknowledgments

The authors thank Editage (www.editage.jp) for English language editing and Ms Satoko Fukaya, who works at our hospital library.

## Author contributions

**Conceptualization:** Seito Shimizu, Hitoshi Hara.

**Writing – original draft:** Seito Shimizu.

**Data curation:** Hitoshi Hara, Yasuhide Muto, Tomoki Kido, Ryohei Miyata.

**Supervision:** Hitoshi Hara.

**Visualization:** Hitoshi Hara.

**Writing – review & editing:** Hitoshi Hara.

## References

[1] Vaez-Zadeh K Dutz W Nowrooz-Zadeh M. Volvulus of the small intestine in adults: a study of predisposing factors. Ann Surg. 1969;169:265–71. 5764212 10.1097/00000658-196902000-00014 PMC1387319

[2] Feng ST Chan T Sun CH. Multiphasic MDCT in small bowel volvulus. Eur J Radiol. 2010;76:e13–8. 19926241 10.1016/j.ejrad.2009.10.026

[3] Otani K Ishihara S Nozawa H. A retrospective study of laparoscopic surgery for small bowel obstruction. Ann Med Surg (Lond). 2017;16:34–9. 28316782 10.1016/j.amsu.2017.02.045 PMC5342981

[4] Roggo A Ottinger LW. Acute small bowel volvulus in adults. A sporadic form of strangulating intestinal obstruction. Ann Surg. 1992;216:135–41. 1503517 10.1097/00000658-199208000-00003 PMC1242584

[5] Hamedani H Nelson B Pagur P Bullmaster J. Spontaneous resolution of symptomatic secondary small bowel volvulus during pre-operative single contrast upper gastrointestinal study. Radiol Case Rep. 2022;17:1810–6. 35369541 10.1016/j.radcr.2022.02.078 PMC8968202

[6] Bauman ZM Evans CH. Volvulus. Surg Clin North Am. 2018;98:973–93. 30243456 10.1016/j.suc.2018.06.005

[7] Barberi C Colaizzi C Guerrini J Kurihara H. Whirl sign: a common misinterpreted radiological entity. Intern Emerg Med. 2021;16:1703–5. 33386605 10.1007/s11739-020-02571-1

[8] Gupta S Mundasad B. Chylous ascites associated with small bowel volvulus: case report on a laparoscopic diagnosis. Middle East J Dig Dis. 2023;15:139–40. 37546511 10.34172/mejdd.2023.334 PMC10404085

[9] Byrnes KG Cullivan O Walsh D Coffey JC. The development of the mesenteric model of abdominal anatomy. Clin Colon Rectal Surg. 2022;35:269–76. 35966981 10.1055/s-0042-1743585 PMC9365479

[10] Islam S Hosein D Dan D Naraynsingh V. Volvulus of ileum: a rare cause of small bowel obstruction. BMJ Case Rep. 2016;2016:bcr2016216159. 10.1136/bcr-2016-216159 PMC5030596 27646320

[11] Hayama T Shioya T Hankyo M. Primary volvulus of the small intestine exhibiting chylous ascites: a case report. J Nippon Med Sch. 2017;84:83–6. 28502964 10.1272/jnms.84.83

[12] Tsang CLN Joseph CT De Robles MSB Putnis S. Primary small bowel volvulus: an unusual cause of small bowel obstruction. Cureus. 2019;11:e6465. 32025394 10.7759/cureus.6465 PMC6977574

[13] Agrawal S Yadav AR Nepal B Upadhyay PK. Primary ileal volvulus: a rare twist in an elderly patient-case report. BMC Surg. 2020;20:237. 33054817 10.1186/s12893-020-00901-w PMC7556909

[14] Leaning M. Chylous ascites as a sequelae of primary small bowel volvulus in a virgin abdomen. J Surg Case Rep. 2021;2021:rjab176. 33981409 10.1093/jscr/rjab176 PMC8104944

[15] Bouassida M Beji H Chtourou MF Ben Othmane N Hamzaoui L Touinsi H. Primary small bowel volvulus: a case report and literature review. Ann Med Surg (Lond). 2022;80:104250. 36045801 10.1016/j.amsu.2022.104250 PMC9422278

[16] Ruiz-Tovar J Morales V Sanjuanbenito A Lobo E Martinez-Molina E. Volvulus of the small bowel in adults. Am Surg. 2009;75:1179–82. 19999908

[17] Tam A Phong J Yong C. Primary small bowel volvulus: surgical treatment dilemma. ANZ J Surg. 2019;89:1521–3. 30562836 10.1111/ans.14967

